# A Retrospective Database Study of Lyme Borreliosis Incidence in Poland from 2015 to 2019: A Public Health Concern

**DOI:** 10.1089/vbz.2022.0049

**Published:** 2023-04-12

**Authors:** Iwona Paradowska-Stankiewicz, Jakub Zbrzeźniak, Jozica Skufca, Archana Nagarajan, Paulina Ochocka, Andreas Pilz, Andrew Vyse, Elizabeth Begier, Mendwas Dzingina, Maxim Blum, Margarita Riera-Montes, Bradford D. Gessner, James H. Stark

**Affiliations:** ^1^ National Institute of Public Health–National Institute of Hygiene–National Research Institute, Department of Infectious Disease Epidemiology and Surveillance, Warsaw, Poland.; ^2^ P95 Epidemiology and Pharmacovigilance, Leuven, Belgium.; ^3^ Pfizer Global Medical Affairs, Vaccines, Vienna, Austria.; ^4^ Patient and Health Impact, Pfizer R&D UK LTD, Walton Oaks, United Kingdom.; ^5^ Vaccine Clinical Research and Development, Pfizer Inc, Pearl River, New York, USA.; ^6^ Vaccine Medical Development & Scientific/Clinical Affairs, Pfizer Inc, Collegeville, Pennsylvania, USA.

**Keywords:** Lyme borreliosis, Lyme arthritis, Poland, 2015–2019, surveillance, epidemiology

## Abstract

**Background:**

In Poland, Lyme borreliosis (LB) has been subject to mandatory public health surveillance since 1996 and, in accordance with EU regulations, Lyme neuroborreliosis has been reported to the European Centre for Disease Prevention and Control since 2019. In this study, the incidence, temporal trends, and geographic distribution of LB and its manifestations in Poland are described for the period 2015–2019.

**Methods:**

This retrospective incidence study of LB and its manifestations in Poland was based on data sent to the National Institute of Public Health–National Institute of Hygiene–National Research Institute (NIPH-NIH-NRI) by district sanitary epidemiological stations using the electronic Epidemiological Records Registration System and data from the National Database on Hospitalization. Incidence rates were calculated using population data from the Central Statistical Office.

**Results:**

During 2015–2019, Poland reported 94,715 cases of LB with an overall average incidence of 49.3 cases per 100,000 population. Cases increased from 2015 (11,945) to 2016 (20,857) and then remained stable through 2019. Hospitalization due to LB also rose during these years. LB was more common among women (55.7%). Erythema migrans and Lyme arthritis were the most common manifestations of LB. The highest incidence rates occurred among >50-year-olds, with a peak in 65–69-year-olds. The highest number of cases was recorded in the third and fourth quarters of the year (July–December). Incidence rates in the eastern and northeastern regions of the country were higher than the national average.

**Conclusions:**

LB is endemic in all regions of Poland, and many regions reported high incidence rates. Large variations in spatially granular incidence rates highlight the need for targeted prevention strategies.

## Introduction

Lyme borreliosis (LB) is an infectious tick-borne disease caused by the bacterial spirochete *Borrelia burgdorferi* sensu lato. It manifests in various forms, such as erythema migrans (EM), and more severe disseminated forms, including Lyme neuroborreliosis (LNB) and Lyme arthritis (LA) (Cardenas-de la Garza et al. 2019). LB has become a public health challenge because of an expanding geographic distribution and increasing incidence attributable to expansion in the range of ticks and animal reservoirs and individual risk behaviors that put humans in contact with ticks (Mannelli et al. 2012).

LB has been subject to mandatory public health surveillance in Poland since 1996. The surveillance system is passive, case based, and involves general practitioners (GPs) and laboratories reporting suspected or confirmed cases of LB to the local and regional health authorities, which in turn notify the National Institute of Public Health–National Institute of Hygiene–National Research Institute (NIPH-NIH-NRI), the authority responsible for processing and archiving data on infectious diseases in Poland.

National case definitions were implemented in 2005 based on definitions from the U.S. Centers for Disease Control and Prevention and the European Union Concerted Action on Lyme Borreliosis (Stanek et al. 1996, US Centers for Disease Control and Prevention, 2021) and were adapted to the Polish surveillance system (Stanek et al. 2011, Andreychyn et al. 2017). Thus, the diagnosis is based on clinical symptoms, history of tick bites, and serological testing. The GPs and laboratories report EM, LNB, LA, and other late manifestations of LB.

LB is considered endemic in Poland, and the number of cases reported has steadily increased since 1996 when surveillance started (Paradowska-Stankiewicz and Chrześcijańska 2014). Incidence rates have more than doubled from 2005 (11.6 LB cases per 100,000 inhabitants) to 2015 (35.4 LB cases per 100,000 inhabitants) (Andreychyn et al. 2017). Even though most voivodeships (highest administrative division in Poland and corresponding to NUTS2 regions) in Poland reported LB cases, the northeastern voivodeships of Podlaskie and Warminsko-Mazurskie recorded rates higher than the national incidence rates, 43.2 and 45.5/100,000 population, respectively, in 2016 (Kozłowski et al. 2021).

This study sought to estimate the incidence of LB nationally and in subnational geographic areas of Poland overall, as well as with respect to various demographic factors, with the most recent available data from 2015 to 2019. These up-to-date data can determine whether the increasing trends have continued and can help inform current and future public health prevention policies.

## Materials and Methods

### Data sources and variables

This retrospective study of LB incidence in Poland from 2015 to 2019 was performed using data from the national surveillance system database maintained by NIPH-NIH-NRI, which has mandatory electronic case-based records of LB and data from the National Database on Hospitalization, prepared as per the mandatory hospitalization reporting by hospitals (ICD-10 codes).

Laboratories or physicians notify LB cases (EM, LNB, LA, and other disseminated forms) to the district Sanitary Epidemiological Stations (SESs), who subsequently register the LB cases in the Epidemiological Records Registration System (ERRS). In addition, district SESs send aggregated data to the appropriate voivodeship SESs, who merge the data from all districts and report to NIPH-NIH-NRI.

Data reported to the ERRS and by voivodeship SESs are compared and collated by NIPH-NIH-NRI, and the LNB cases have been reported to TESSy (The European Surveillance System) since 2019 (Supplementary Fig. S1). The case definitions used for the diagnosis of LB and various manifestations of LB are summarized in Supplementary Table S1. All the cases are classified into two categories: probable and confirmed.

To investigate the geographic distribution, demographic characteristics, and seasonality of LB, data were extracted for the period January 1, 2015, to December 31, 2019, from the ERRS. In this study, data on cases of LB were extracted and stratified by year, geographic areas (voivodeships, NUTS2), week of notification, sex, age, and three case definition categories (suspected, probable, and confirmed). The incidence rate was then calculated as the number of cases divided by the corresponding population size and multiplied by 100,000.

All population size data (by voivodeship, age, and sex) were extracted from the Central Statistical Office website (Central Statistical Office [Poland], 2022). The average number of cases and the average incidence over the 5-year period (2015–2019) were calculated as the mean of the annual number of cases to the mean population size.

This review is based on previously conducted studies and does not contain any new studies with human participants or animals performed by any of the authors; therefore, Institutional Review Board approval was not required.

## Results

### Time trend and LB manifestations

**FIG. 1.**
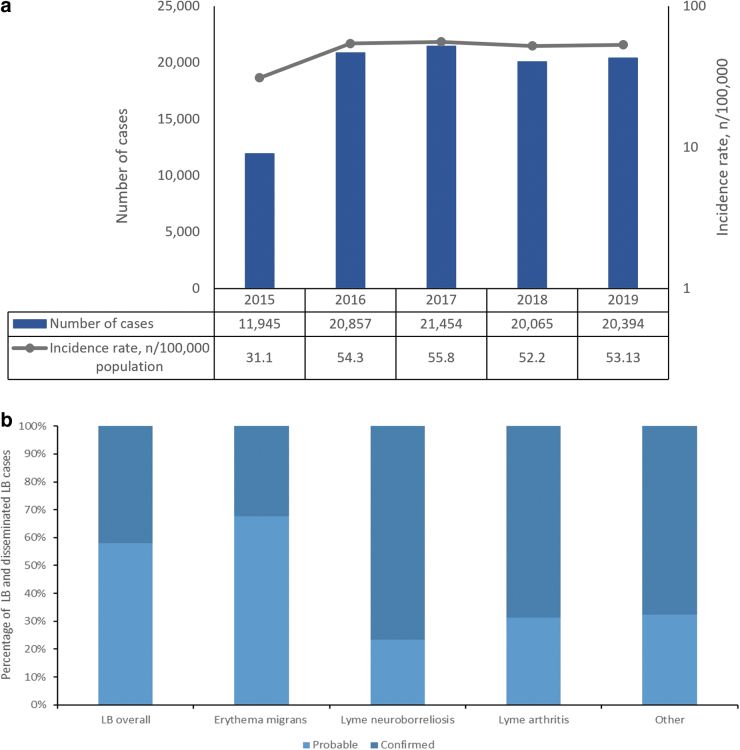
**(a)** Number of cases and annual incidence rates of LB. **(b)** Percentage of LB and disseminated cases according to the level of diagnostic certainty (probable and confirmed) in Poland, 2015–2019. LB, Lyme borreliosis.

Poland reported 94,715 cases of LB (including various manifestations) from 2015 to 2019, ranging from 11,945 cases in 2015 to 21,454 cases in 2017 (Table 1 and Fig. 1a). The overall mean LB incidence rate for 2015–2019 was 49.3 per 100,000 population (Table 1).

**Table 1.** Average Incidence Rate of Lyme Borreliosis by Disease Manifestation, 2015–2019

LB manifestation	No. of cases	Average annual incidence rate/100,000 population	LB manifestation*^a^* (%)
Erythema migrans	69,581	36.2	73.5
Lyme neuroborreliosis	1608	0.8	1.7
Lyme arthritis	30,330	15.8	32.0
Other overall^b^	2370	1.2	2.5
Other—ACA	56	0.03	0.1
Other—Lyme carditis	160	0.1	0.2
Other—Borrelial lymphocytoma	97	0.1	0.1
Overall LB	94,715	49.3	100.0^c^

^a^ Many cases reported more than one manifestation.

^b^ Other overall includes ACA, Lyme carditis, and Borrelial lymphocytoma, as well as any other case of LB, which could not be further characterized.

^c^ 100% represents overall number of cases of LB, but is not a sum of the above-mentioned percentages.

ACA, acrodermatitis chronica atrophicans; LB, Lyme borreliosis.

The most common manifestations of LB were EM and LA, with incidence rates of 36.2 and 15.8 per 100,000 population, respectively. EM and LA accounted for ∼74% and 32% of all manifestations of LB, respectively. LNB had an incidence rate of 0.8 per 100,000 population and accounted for 1.7% of all LB cases.

Other manifestations (acrodermatitis chronica atrophicans [ACA], Lyme carditis, and Borrelial lymphocytoma) accounted for ∼2.5% of LB cases (Table 1). Some patients also reported more than one manifestation. A total of 8347 cases reported both EM and LA from 2015 to 2019 (Supplementary Table S2).

**FIG. 2.**
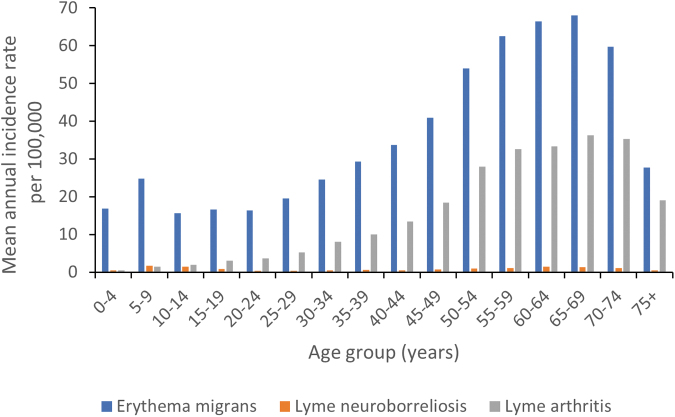
The mean annual incidence rates of various manifestations of LB among different age groups.

The analysis of LB by level of diagnostic certainty (probable and confirmed cases) showed that the percentage of probable cases was higher for overall LB and EM compared with the disseminated LB manifestations (Fig. 2). Although laboratory confirmation is not required for diagnosing EM manifestations (unlike disseminated manifestations, which do require laboratory confirmation), about 30% of EM cases had evidence of laboratory confirmation.

### Demographic and socioeconomic characteristics

LB was more common among women (55.7% of all LB cases and incidence rate of 53.2 per 100,000 population). Women constituted 56.5% of EM, 55.6% of LA, and 42.4% of LNB cases, with incidence of 39.6, 17.0, and 0.7 per 100,000 population, respectively (Supplementary Table S3).

Higher incidence rates were reported in the population aged between 50 and 74 years for the period 2015–2019. The highest mean annual incidence rate (96.1 per 100,000 population) was among persons 65–69 years old (Supplementary Table S4). Among the population <18 years old, the highest mean annual incidence rate was observed in 5–9-year-olds (28.0 per 100,000 population).

EM incidence rates peaked in persons aged 60–64 and 65–69 years, while the peak LA incidence rates were observed in persons aged 65–69 and 70–74 years (Fig. 2).

### Seasonality

**FIG. 3.**
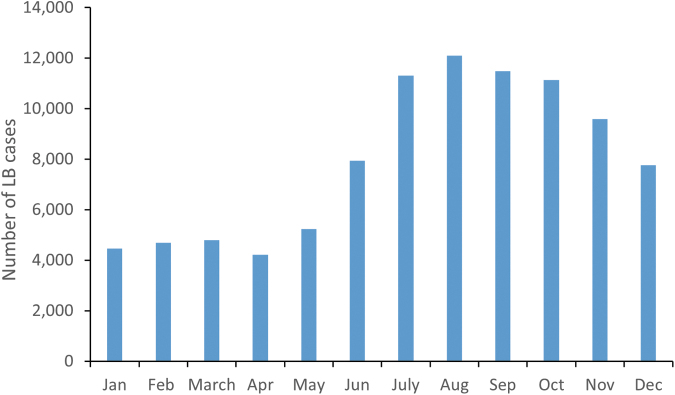
Number of LB cases by month during 2015–2019.

LB occurred all year round, but with a seasonal pattern, with higher incidence from June to November. The highest monthly proportion of LB cases was reported in August (12.8% of the annual number of cases) and September (12.1% of all cases). Most cases were reported in the third quarter of the year, followed by the fourth quarter (Fig. 3).

### Geographic distribution

**FIG. 5.**
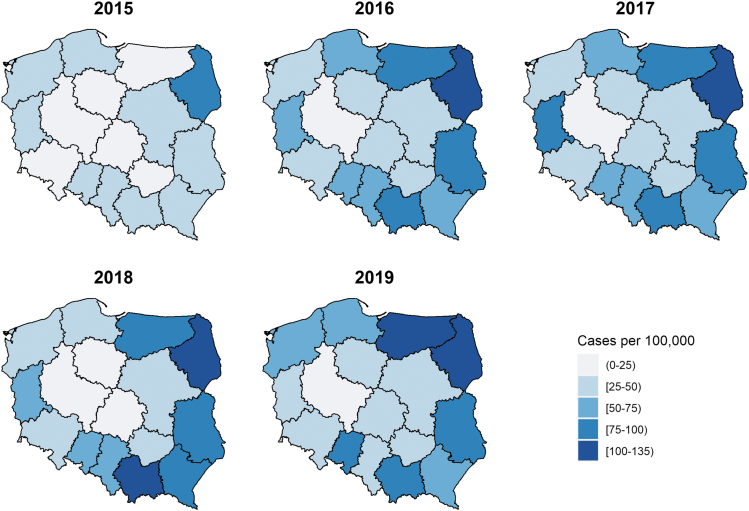
The annual LB incidence rate (number of cases per 100,000 population) across voivodeships in Poland from 2015 to 2019.

During 2015–2019, LB in Poland was characterized by high incidence rates in the east and northeast of the country (Podlaskie, Lubelskie, and Warmińsko-Mazurskie voivodeships), where the average annual incidence of LB ranged from 78.8 per 100,000 population in the Warmińsko-Mazurskie voivodeship to 115.2 per 100,000 population in the Podlaskie voivodeship. Małopolskie voivodeship in the south also had a high average incidence rate (86.4 per 100,000) (Fig. 5).

In the voivodeships of Lubelskie (east of Poland) and Lubuskie (west Poland), the incidence rates almost doubled from 2015 to 2016 and stayed high thereafter, except for Lubuskie in 2019 when it again dropped below 50 (Supplementary Table S5). The voivodeships in central Poland had the lowest incidence rates. Wielkopolskie and Lodskie had average annual incidence rates of 13.7 and 24.8 per 100,000 population, respectively, which were below the national average of 49.3 per 100,000 persons (Table 1 and Fig. 5).

### LB hospitalizations

From 2015 to 2019, there were a total of 48,717 hospitalizations with LB diagnosis (range: 7717 in 2015 to 10,718 in 2018); however, the number of persons hospitalized with LB is unknown since some persons could have been hospitalized more than once. The highest numbers of LB hospitalizations were reported among persons aged 55–59, 60–64, and 65–69 years, corresponding with high overall LB incidence rates in these age groups.

LB hospitalizations were consistent during the study period, except in 2015 when overall LB hospitalizations were comparatively lower (Supplementary Fig. S2A). Age-specific distribution of hospitalizations in males and females reflected the overall LB hospitalization trend. The only difference was the higher number of hospitalizations in 70–74- and >75-year-old females (Supplementary Fig. S2B, C).

## Discussion

This retrospective study in Poland and its regions, using data from NIPH-NIH for 2015–2019, described changes in LB incidence nationally and in geographic subareas. Poland reported 94,715 LB cases across this period and had a high burden of disease, with an average overall annual incidence rate of 49.3 per 100,000 population from 2015 to 2019.

LB has continued to increase throughout the country and is now endemic in all regions of Poland, with overall annual incidence rates ranging from 31.1 (2015) to 55.8 (2018) per 100,000 population. The lower incidence rates in 2015 compared with 2016 may be due to change in the case definition from a one-tier test (Western blot) before 2015 to two-tier tests (ELISA and Western blot) since then. This stricter definition led to a drop in numbers in 2015.

However, testing in Poland is paid for privately, and many people still get only one test done. Thus, the numbers reported from 2016 rose again as one-tier results (ELISA or Western blot) were included. However, Western blot should not be used as a stand-alone test, against the recommended two-tier tests, as the choice of antigens and interpretation of results can vary, leading to false positives and thus may result in an overestimation of LB case numbers (Dessau et al. 2018).

The increase in the number of reported cases might also be attributed to increased awareness among citizens and physicians (Gutknecht et al. 2019). In a survey conducted by Gutknecht et al., the GPs in Pomerania were found to be highly knowledgeable about LB and most often diagnosed LB correctly (Gutknecht et al. 2019).

The average annual national incidence rate of 49.3 per 100,000 inhabitants is higher than that of the neighboring countries, Czech Republic (37.3/100,000 population between 2007 and 2016), Germany (33/100,000 between 2013 and 2017), and Belarus (25.5/100,000 in 2019) (Enkelmann et al. 2018, Kříž et al. 2018, Kniazeva et al. 2021).

By contrast, Polish incidence rates were lower than those reported from other nearby European countries such as Finland (99.6/100,000 between 2015 and 2020, *pers.comm*) and Lithuania (85.4/100,000 between 2014 and 2016) (Petrulionienė et al. 2020). Comparison among European countries is complicated and limited by differences in surveillance systems (mandatory vs. voluntary) and varying case definitions for LB, which rely on different diagnostic testing strategies to confirm a case (Rizzoli et al. 2011).

**FIG. 4.**
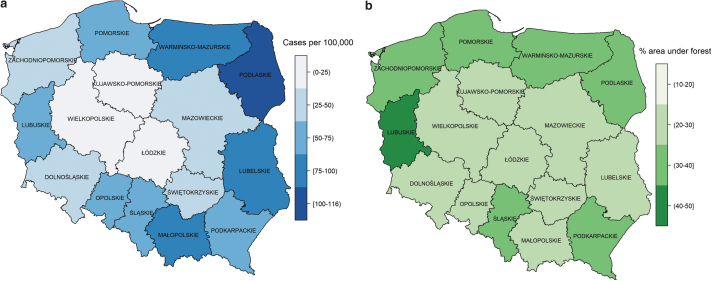
**(a)** Average incidence rate of LB by voivodeship in Poland. **(b)** Forest cover in Poland in 2015 (Central Statistical Office 2016).

High incidence occurred in eastern Poland (Fig. 4a). Two of the high incidence areas—Podlaskie and Warmińsko-Mazurskie—have 30.3% of forest cover (Fig. 4b) and share borders with the neighboring country of Lithuania, which has one of the highest LB incidence rates in Europe. Lubuskie, in the west of Poland, with the highest proportion of forest cover (∼49%), doubled its incidence rate between 2015 and 2016, and it has remained high since then (Fig. 5).

Forests present an ideal environment for ticks, which require high humidity, vegetation, and the presence of animal reservoirs (Kilpatrick et al. 2017). Furthermore, agricultural lands also account for large areas in Podlaskie and could contribute to the high incidence rates in this voivodeship (Sovkich et al. 2017).

The urban population also seems to be increasingly affected by LB in Poland, with urban parks and gardens reporting high tick activity (Kozłowski et al. 2021). Buczek et al. (2014) conducted a study on the risk of LB in southwest Poland and found that the phenomenon of “urban heat islands”—wherein there are daily variations in temperature and humidity due to prevailing dust and pollution—might have caused an increase in tick density and prevalence of LB. In this study, the cities of Lublin and Bialystok in eastern Poland and the cities of Krakow and Katowice in southern Poland also had relatively higher LB incidence.

More cases of LB were reported among females than males. The reason for this is unclear. However, this trend has also been observed in many regions and countries of Europe (Wilking and Stark 2014, Sajanti et al. 2017, Petrulionienė et al. 2020). Age distribution of the incidence presented a bimodal curve with peaks in the 5–9- and 50–74-year age groups, as reported in other countries, although the peak in the 5–9-year age group was less pronounced (Gasmi et al. 2017, Sajanti et al. 2017, Schwartz et al. 2017).

The higher incidence in people aged 50–74 years may be related to the professions of people aged 50–65 years (*e.g.*, agriculture, forestry). There have been many studies in the eastern and southern parts of Poland showing higher seroprevalence and risk of LB in forestry workers and hunters (Buczek et al. 2009, Cisak et al. 2012, Lewandowska et al. 2013). Higher incidence among older persons and females could also be related to leisure activities of gardening and walking and mushroom/berry picking in forests among these populations.

The most common LB manifestations were EM and LA among various age groups. High incidence of LA cases may be attributed to the predominant *Borrelia* genospecies. The three predominant *Borrelia* genospecies found on ticks and patients with LB in Poland are *B. burgdorferi* sensu stricto, *B. garinii*, and *B. afzelii* (Kubiak et al. 2012, Grygorczuk et al. 2013, Kiewra and Zaleśny 2013, Strzelczyk et al. 2015), with the former being particularly associated with LA.

The very limited data available in Poland indicate that in contrast to most other European countries, *B. burgdorferi* sensu stricto is potentially the most predominant species, with infected ticks having a prevalence of 61.4% (Strzelczyk et al. 2015) and with the majority of 259 LB patients (71.5%) presenting IgM antibodies specific for the flagellin protein of *B. burgdorferi* sensu stricto (Kubiak et al. 2012).

People reporting both EM and LA from 2015 to 2019 (8347 cases) constituted almost one-third of the LA cases reported. This could be an indication of overdiagnosis of LA, which might be influenced by other factors, such as age. Furthermore, the probable case definition for LA does not include laboratory confirmation as in the confirmed case definition.

Laboratory diagnostics for LA have high sensitivity and specificity; therefore, the probable cases reported may not reflect a true LA case (Branda and Steere 2021). Nevertheless, the proportion of cases by each manifestation is likely to vary between countries due to reporting procedures, testing, health care professionals and public awareness, health care utilization and access, and the predominant circulating *Borrelia* genospecies; therefore, comparing the Poland data with other countries is not recommended.

Distinct seasonality was observed in LB cases, with a peak in cases in the third quarter of the year. This can be attributed to the increased tick activity in spring and summer in Poland (Kiewra and Lonc 2004, Zając et al. 2021). In addition, a high number of LB cases were recorded through November, presumably from increased activities in the fall, such as mushroom picking. However, a substantial number of cases also occurred throughout the year, which stresses the need for year-round prevention strategies.

### Limitations

Differences exist in LB classification among GPs, sanitary stations, and the NIPH-NIH-NRI. This is due to differences in clinical diagnosis by doctors, the variety of serological tests, and the difficulty in diagnosing some manifestations, such as LNB. LB skin manifestations (EM, ACA, and Borrelial lymphocytoma) may not always be reported or recognized, resulting in an underestimation of LB incidence.

The recommendation of a two-stage diagnosis of LB has not yet been incorporated into standard practice. The changes to the database system might have impacted data quality. In recent years, ERRS was replaced by EPiBaza in Poland, and thus data analysis required matching databases with different structures to achieve compatibility.

## Summary and Conclusions

The number of cases and incidence of LB have been on the rise in Poland since mandatory notification came into place, and cases have remained stable in recent years (2016–2019). Compared with areas with low incidence rates, incidence rates were ninefold higher in the east and northeast of the country (Podlaskie, Lubelskie, and Warmińsko-Mazurskie voivodeships), which corresponded to regions with more forest cover and large agricultural areas. Persons aged 50–74 years had higher incidence compared with other age groups.

These results emphasize the need for data that are spatially granular and age specific to guide LB prevention strategies. Furthermore, clarity on manifestation reporting and *Borrelia* species testing will provide a richer picture of the disease burden in Poland.

## Supplementary Material

Supplemental data

Supplemental data

Supplemental data

Supplemental data

Supplemental data

Supplemental data

Supplemental data
