# Supplementary material for: A Retrospective Database Study of Lyme Borreliosis Incidence in Poland from 2015 to 2019: A Public Health Concern
Source: Vector Borne Zoonotic Dis. 2023 Apr 12;23(4):247–55. doi: 10.1089/vbz.2022.0049 (PMC10122228; doi:10.1089/vbz.2022.0049)
Supplement: Supplemental data [file Supp_FigS1.docx]

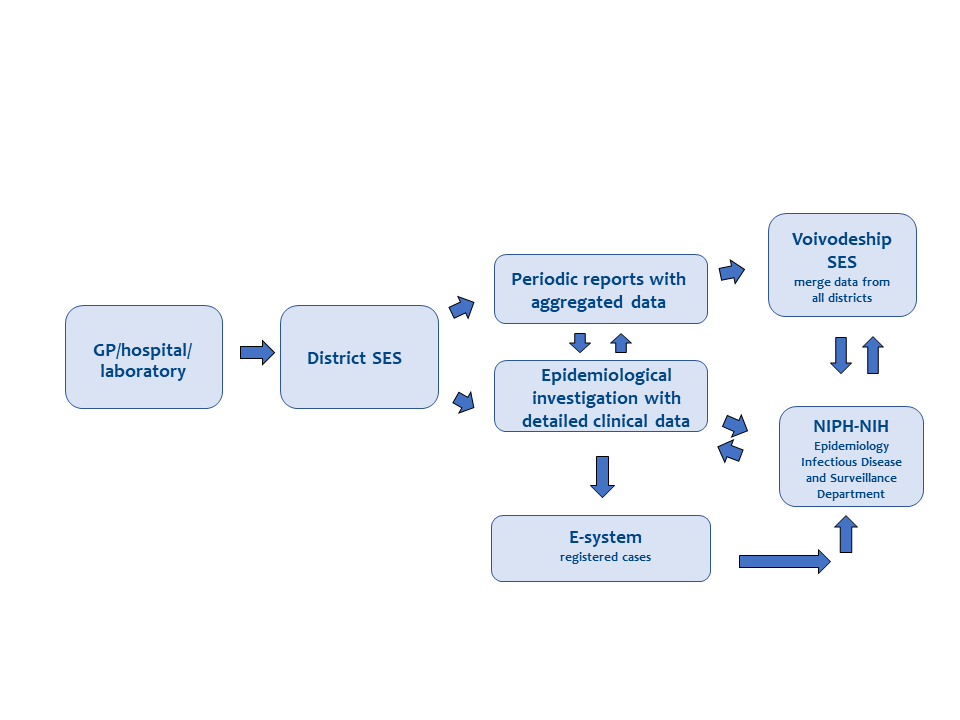


**Supplementary Figure 1:** Surveillance and reporting of LB in Poland. GP: General Practitioners; SES: Sanitary Epidemiological Stations; NIPH NIH- NRI -National Institute of Public Health National Institute of Hygiene – National Research Institute
