# Supplementary material for: A Retrospective Database Study of Lyme Borreliosis Incidence in Poland from 2015 to 2019: A Public Health Concern
Source: Vector Borne Zoonotic Dis. 2023 Apr 12;23(4):247–55. doi: 10.1089/vbz.2022.0049 (PMC10122228; doi:10.1089/vbz.2022.0049)
Supplement: Supplemental data [file Supp_TableS1.docx]

**Supplementary Table 1:** Case definitions

| **Diagnosis** | **Case definition** |
| --- | --- |
| **Lyme borreliosis (LB)*** | |
| Probable case | ICD for LB diagnosis (ICD-10 code A69.2)  **OR**  LB diagnosis in free text in record  **AND, EITHER [**  equivocal or unspecified laboratory tests results for LB  **OR**  LB appropriate antibiotics (e.g. phenoxymethylpenicillin, doxycycline, amoxicillin,) within ± 14 days of incident date **]** |
| Confirmed case | **[**Probable case **]**  **AND**  laboratory confirmation, **EITHER [**  A positive culture or PCR for *B. burgdorferi*  **OR**  A positive two-tier test: positive ELISA test within ± 180 days of incident date AND positive Western Blot within ± 180 days of index date  **OR**  A positive single-tier Western Blot within ± 90 days of index date**]** |
| **Erythema migrans (EM)** | |
| Probable case | EM diagnosis in free text in record  **AND, EITHER [**  Equivocal or unspecified laboratory tests results for EM  **OR**  EM appropriate antibiotics (e.g. phenoxymethylpenicillin, doxycycline, amoxicillin, cefuroxyme axetil, azithromycin) within ± 14 days of incident date**]** |
| Confirmed case | **[**Probable case **]**  **AND**  laboratory confirmation, **EITHER [**  A positive culture or PCR for *B. burgdorferi*  **OR**  A positive two-tier test: positive ELISA test within ± 180 days of index date AND positive Western Blot within ± 180 days of index date  **OR**  A positive single-tier Western Blot within ± 90 days of incident date |
| **Disseminated Lyme borreliosis: Lyme arthritis (LA)** | |
| Probable case | Findings compatible with LA and associated with a LB ICD code (ICD-10 A69.2)  **AND**  LB or EM appropriate antibiotics (e.g. phenoxymethylpenicillin, doxycycline, amoxicillin, ceftriaxone) within ± 14 days of index date**]**  **OR**  Arthritis associated with LB and with a specific ICD code (e.g. ICD-10 A69.2) |
| Confirmed case | **[**Probable case**]**  **AND**  laboratory confirmation through positive two-tier serologic tests |
| **Disseminated Lyme borreliosis: Lyme neuroborreliosis (LNB)** | |
| Probable case | Findings compatible with LNB and associated with a LB specific ICD or other code (e.g. A69.2 in combination with G01.9 for meningitis in bacterial diseases classified elsewhere, Annex 2)  **AND EITHER [**  LB or EM appropriate antibiotics (e.g. phenoxymethylpenicillin, doxycycline, amoxicillin, ceftriaxone) within ± 14 days)  **OR**  pleocytosis in the cerebrospinal fluid (CSF)  **OR**  a positive serology in CSF  **OR**  a positive CSF–serum index, without pleocytosis and neurological findings**]** |
| Confirmed case | **[**Probable case **]**  **AND** laboratory confirmation, **EITHER [**  A pleocytosis in CSF and intrathecal antibody production (antibody index, AI), determined by paired positive serology (ELISA and Western blot) in serum and in CSF in samples obtained on the same day  **OR**  positive PCR on CSF **]** |
| **Other manifestations (OTH) ACA, LC, Borrelial lymphocytoma** | |
| Probable case | Based on selection of OTH by area and availability of data, e.g. a person visiting healthcare provider for acrodermatitis chronica atrophicans (ACA) or Lyme carditis or Borrelial lymphocytoma associated with a LB disease code ICD-10 code A69.2  **AND, EITHER** **[**  LB or EM appropriate antibiotics (e.g. phenoxymethylpenicillin, doxycycline, amoxicillin, ceftriaxone) within ± 14 days)  **OR**  positive serology**]** |
| Confirmed case | **[**Probable case**]**  **AND** laboratory confirmation,  **EITHER [**  Positive culture and positive skin PCR  **OR**  suggestive histopathologic findings] |

*LB diagnosis in free text record (including keywords covering all manifestations of LB such as erythema migrans, lyme arthritis, lyme neuroborreliosis, ACA).
