# Supplementary material for: A Retrospective Database Study of Lyme Borreliosis Incidence in Poland from 2015 to 2019: A Public Health Concern
Source: Vector Borne Zoonotic Dis. 2023 Apr 12;23(4):247–55. doi: 10.1089/vbz.2022.0049 (PMC10122228; doi:10.1089/vbz.2022.0049)
Supplement: Supplemental data [file Supp_TableS2.docx]

**Supplementary Table 2:** Number of cases that reported more than one LB manifestations, 2015-2019

| **Type of manifestations** | **Number of cases** |
| --- | --- |
| Erythema migrans and Lyme arthritis | 8,347 |
| Erythema migrans and Lyme neuroborreliosis | 245 |
| Lyme neuroborreliosis and Lyme arthritis | 183 |
| Erythema migrans, Lyme neuroborreliosis and Lyme arthritis | 41 |
| Lyme neuroborreliosis and other manifestations | 11 |
| Erythema migrans and other manifestations | 134 |
| Lyme arthritis and other manifestations | 111 |
| Erythema migrans, Lyme neuroborreliosis, Lyme arthritis and other | 102 |
