# Supplementary material for: A Retrospective Database Study of Lyme Borreliosis Incidence in Poland from 2015 to 2019: A Public Health Concern
Source: Vector Borne Zoonotic Dis. 2023 Apr 12;23(4):247–55. doi: 10.1089/vbz.2022.0049 (PMC10122228; doi:10.1089/vbz.2022.0049)
Supplement: Supplemental data [file Supp_TableS3.docx]

**Supplementary Table 3.** Number of cases and incidence rate/100,000 population of LB manifestations by sex in Poland, 2015-2019

| LB manifestation | Number of cases | | Average annual incidence rate/100,000 inhabitants | |
| --- | --- | --- | --- | --- |
|  | *Male* | *Female* | *Male* | *Female* |
| Lyme borreliosis overall | 41,960 | 52,755 | 45.2 | 53.2 |
| Erythema migrans (EM) | 30,277 | 39,304 | 32.6 | 39.6 |
| Lyme arthritis (LA) | 13,461 | 16,869 | 14.5 | 17.0 |
| Lyme neuroboreliosis (LNB) | 926 | 682 | 1.0 | 0.7 |
