# Supplementary material for: A Retrospective Database Study of Lyme Borreliosis Incidence in Poland from 2015 to 2019: A Public Health Concern
Source: Vector Borne Zoonotic Dis. 2023 Apr 12;23(4):247–55. doi: 10.1089/vbz.2022.0049 (PMC10122228; doi:10.1089/vbz.2022.0049)
Supplement: Supplemental data [file Supp_TableS4.docx]

**Supplementary Table 4:** Lyme borreliosis incidence rate by age group (per 100,000 inhabitants) in Poland, 2015-2019.

| Year | 2015 | 2016 | 2017 | 2018 | 2019 | Mean |
| --- | --- | --- | --- | --- | --- | --- |
| Age Group |  |  |  |  |  |  |
| 0-4 | 9.4 | 17.6 | 19.8 | 20.0 | 21.3 | 17.6 |
| 5-9 | 13.7 | 30.8 | 30.2 | 33.0 | 32.5 | 28.0 |
| 10-14 | 12.2 | 20.2 | 21.8 | 20.1 | 20.3 | 18.9 |
| 15-19 | 12.8 | 23.3 | 22.4 | 22.0 | 22.2 | 20.5 |
| 20-24 | 13.8 | 22.9 | 21.6 | 20.3 | 21.5 | 20.0 |
| 25-29 | 16.1 | 28.6 | 25.1 | 24.4 | 27.5 | 24.3 |
| 30-34 | 19.6 | 35.3 | 36.7 | 33.0 | 34.6 | 31.9 |
| 35-39 | 22.5 | 43.9 | 41.6 | 39.7 | 41.0 | 37.7 |
| 40-44 | 28.5 | 50.0 | 51.1 | 47.6 | 45.7 | 44.6 |
| 45-49 | 36.6 | 64.7 | 64.3 | 55.6 | 57.4 | 55.7 |
| 50-54 | 51.2 | 86.8 | 84.5 | 79.0 | 80.1 | 76.3 |
| 55-59 | 58.2 | 96.0 | 99.5 | 95.2 | 93.8 | 88.5 |
| 60-64 | 60.3 | 100.0 | 107.3 | 98.2 | 97.3 | 92.6 |
| 65-69 | 58.5 | 103.7 | 110.9 | 101.6 | 105.9 | 96.1 |
| 70-74 | 58.9 | 94.9 | 101.5 | 91.5 | 90.0 | 87.4 |
| 75+ | 27.9 | 47.2 | 50.2 | 46.3 | 46.4 | 43.6 |
