# Supplementary material for: A Retrospective Database Study of Lyme Borreliosis Incidence in Poland from 2015 to 2019: A Public Health Concern
Source: Vector Borne Zoonotic Dis. 2023 Apr 12;23(4):247–55. doi: 10.1089/vbz.2022.0049 (PMC10122228; doi:10.1089/vbz.2022.0049)
Supplement: Supplemental data [file Supp_TableS5.docx]

**Supplementary Table 5:** Total number of LB cases by voivodeships and overall per year, 2015-2019.

| **Year** | **Voivodeship** | **No. of cases, n** | **Population** | **Incidence, n/100,000** |
| --- | --- | --- | --- | --- |
| **2015** |  |  |  |  |
|  | DOLNOŚLĄSKIE | 517 | 2,904,207 | 17.80 |
|  | KUJAWSKO-POMORSKIE | 392 | 2,086,210 | 18.79 |
|  | LUBELSKIE | 1,065 | 2,139,726 | 49.77 |
|  | LUBUSKIE | 395 | 1,018,075 | 38.80 |
|  | ŁÓDZKIE | 444 | 2,493,603 | 17.81 |
|  | MAŁOPOLSKIE | 1,651 | 3,372,618 | 48.95 |
|  | MAZOWIECKIE | 1,426 | 5,349,114 | 26.66 |
|  | OPOLSKIE | 384 | 996,011 | 38.55 |
|  | PODKARPACKIE | 784 | 2,127,657 | 36.85 |
|  | PODLASKIE | 1,142 | 1,188,800 | 96.06 |
|  | POMORSKIE | 820 | 2,307,710 | 35.53 |
|  | ŚLĄSKIE | 2,147 | 4,570,849 | 46.97 |
|  | ŚWIĘTOKRZYSKIE | 152 | 1,257,179 | 12.09 |
|  | WARMIŃSKO-MAZURSKIE | 159 | 1,439,675 | 11.04 |
|  | WIELKOPOLSKIE | 2 | 3,475,323 | 0.06 |
|  | ZACHODNIOPOMORSKIE | 465 | 1,710,482 | 27.19 |
|  | **ALL** | 11,945 | 38,437,239 | 31.08 |
| **2016** |  |  |  |  |
|  | DOLNOŚLĄSKIE | 929 | 2,903,710 | 31.99 |
|  | KUJAWSKO-POMORSKIE | 607 | 2,083,927 | 29.13 |
|  | LUBELSKIE | 1,892 | 2,133,340 | 88.69 |
|  | LUBUSKIE | 633 | 1,017,376 | 62.22 |
|  | ŁÓDZKIE | 772 | 2,485,323 | 31.06 |
|  | MAŁOPOLSKIE | 2,899 | 3,382,260 | 85.71 |
|  | MAZOWIECKIE | 2,168 | 5,365,898 | 40.40 |
|  | OPOLSKIE | 670 | 9,93,036 | 67.47 |
|  | PODKARPACKIE | 1,074 | 2,127,656 | 50.48 |
|  | PODLASKIE | 1,589 | 1,186,625 | 133.91 |
|  | POMORSKIE | 1,286 | 2,315,611 | 55.54 |
|  | ŚLĄSKIE | 3,168 | 4,559,164 | 69.49 |
|  | ŚWIĘTOKRZYSKIE | 381 | 1,252,900 | 30.41 |
|  | WARMIŃSKO-MAZURSKIE | 1,375 | 1,436,367 | 95.73 |
|  | WIELKOPOLSKIE | 568 | 3,481,625 | 16.31 |
|  | ZACHODNIOPOMORSKIE | 846 | 1,708,174 | 49.53 |
|  | **ALL** | 20,857 | 38,432,992 | 54.27 |
| **2017** |  |  |  |  |
|  | DOLNOŚLĄSKIE | 851 | 2,902,547 | 29.32 |
|  | KUJAWSKO-POMORSKIE | 544 | 2,082,944 | 26.12 |
|  | LUBELSKIE | 1,974 | 2,126,317 | 92.84 |
|  | LUBUSKIE | 775 | 1,016,832 | 76.22 |
|  | ŁÓDZKIE | 637 | 2,476,315 | 25.72 |
|  | MAŁOPOLSKIE | 3,319 | 3,391,380 | 97.87 |
|  | MAZOWIECKIE | 2,211 | 5,384,617 | 41.06 |
|  | OPOLSKIE | 649 | 990,069 | 65.55 |
|  | PODKARPACKIE | 1,480 | 2,129,138 | 69.51 |
|  | PODLASKIE | 1,535 | 1,184,548 | 129.59 |
|  | POMORSKIE | 1,455 | 2,324,251 | 62.60 |
|  | ŚLĄSKIE | 2,774 | 4,548,180 | 60.99 |
|  | ŚWIĘTOKRZYSKIE | 449 | 1,247,732 | 35.99 |
|  | WARMIŃSKO-MAZURSKIE | 1,298 | 1,433,945 | 90.52 |
|  | WIELKOPOLSKIE | 655 | 3,489,210 | 18.77 |
|  | ZACHODNIOPOMORSKIE | 848 | 1,705,533 | 49.72 |
|  | **ALL** | 21,454 | 38,433,558 | 55.82 |
| **2018** |  |  |  |  |
|  | DOLNOŚLĄSKIE | 849 | 2,901,225 | 29.26 |
|  | KUJAWSKO-POMORSKIE | 369 | 2,077,775 | 17.76 |
|  | LUBELSKIE | 1,951 | 2,117,619 | 92.13 |
|  | LUBUSKIE | 608 | 1,014,548 | 59.93 |
|  | ŁÓDZKIE | 598 | 2,466,322 | 24.25 |
|  | MAŁOPOLSKIE | 3,616 | 3,400,577 | 106.33 |
|  | MAZOWIECKIE | 1,613 | 5,403,412 | 29.85 |
|  | OPOLSKIE | 693 | 986,506 | 70.25 |
|  | PODKARPACKIE | 1,742 | 2,129,015 | 81.82 |
|  | PODLASKIE | 1,287 | 1,181,533 | 108.93 |
|  | POMORSKIE | 1,145 | 2,333,523 | 49.07 |
|  | ŚLĄSKIE | 2,590 | 4,533,565 | 57.13 |
|  | ŚWIĘTOKRZYSKIE | 422 | 1,241,546 | 33.99 |
|  | WARMIŃSKO-MAZURSKIE | 1,297 | 1,428,983 | 90.76 |
|  | WIELKOPOLSKIE | 519 | 3,493,969 | 14.85 |
|  | ZACHODNIOPOMORSKIE | 766 | 1,701,030 | 45.03 |
|  | **ALL** | 20,065 | 38,411,148 | 52.24 |
| **2019** |  |  |  |  |
|  | DOLNOŚLĄSKIE | 888 | 2,900,163 | 30.62 |
|  | KUJAWSKO-POMORSKIE | 563 | 2,072,373 | 27.17 |
|  | LUBELSKIE | 1,843 | 2,108,270 | 87.42 |
|  | LUBUSKIE | 476 | 1,011,592 | 47.05 |
|  | ŁÓDZKIE | 622 | 2,454,779 | 25.34 |
|  | MAŁOPOLSKIE | 3,168 | 3,410,901 | 92.88 |
|  | MAZOWIECKIE | 2,233 | 5,423,168 | 41.18 |
|  | OPOLSKIE | 787 | 982,626 | 80.09 |
|  | PODKARPACKIE | 1,399 | 2,127,164 | 65.77 |
|  | PODLASKIE | 1,266 | 1,178,353 | 107.44 |
|  | POMORSKIE | 1,606 | 2,343,928 | 68.52 |
|  | ŚLĄSKIE | 2,134 | 4,517,635 | 47.24 |
|  | ŚWIĘTOKRZYSKIE | 332 | 1,233,961 | 26.91 |
|  | WARMIŃSKO-MAZURSKIE | 1,513 | 1,422,737 | 106.34 |
|  | WIELKOPOLSKIE | 640 | 3,498,733 | 18.29 |
|  | ZACHODNIOPOMORSKIE | 924 | 1,696,193 | 54.47 |
|  | **ALL** | 20,394 | 38,382,576 | 53.13 |
