# Supplementary material for: A Retrospective Database Study of Lyme Borreliosis Incidence in Poland from 2015 to 2019: A Public Health Concern
Source: Vector Borne Zoonotic Dis. 2023 Apr 12;23(4):247–55. doi: 10.1089/vbz.2022.0049 (PMC10122228; doi:10.1089/vbz.2022.0049)
Supplement: Supplemental data [file Supp_FigS2.docx]

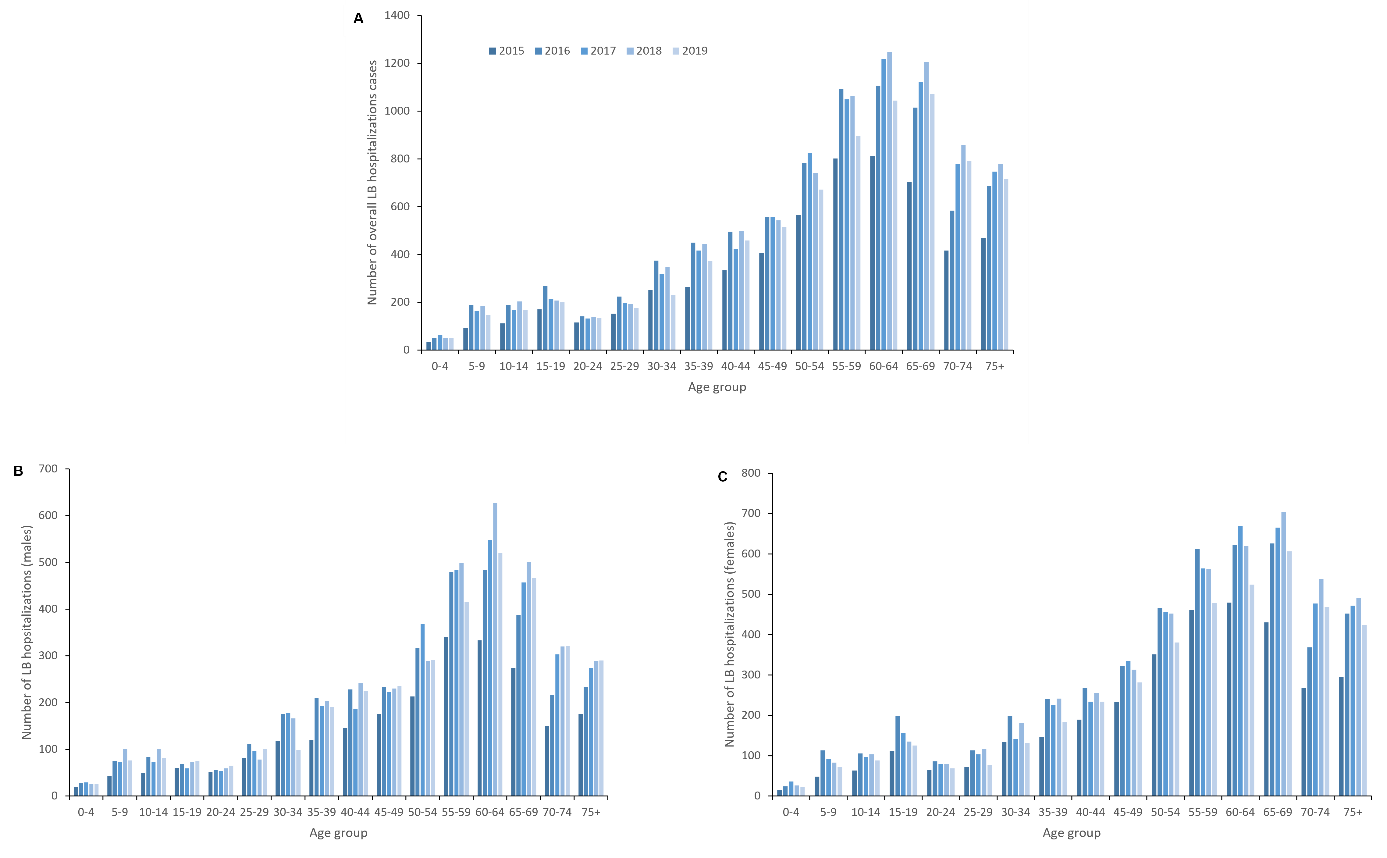


**Supplementary Figure 2**: Lyme borreliosis hospitalizations by age groups (A), in males (B), and in females (C) in Poland in 2015-19
